# Multitask-Based Anti-Collision Trajectory Planning of Redundant Manipulators

**DOI:** 10.3390/biomimetics9110679

**Published:** 2024-11-06

**Authors:** Suping Zhao, Yushuang Du, Chaobo Chen, Xiaohua Song, Xiaoyan Zhang

**Affiliations:** College of Electronic Information Engineering, Xi’an Technological University, Xi’an 710021, China; zhaosuping@xatu.edu.cn (S.Z.); yushuangdu@163.com (Y.D.); songxiaohua@xatu.edu.cn (X.S.); xyzhang@xatu.edu.cn (X.Z.)

**Keywords:** redundant manipulator, multitask-based trajectory planning, special encoding genetic algorithm, anti collision

## Abstract

During performing multiple tasks of a redundant manipulator, the obstacles affect the sequential order of task areas and the joint trajectories. The end-effector is constrained to visit multiple task areas with an optimal anti-collision path, while the joints are required to move smoothly and avoid predefined obstacles. A special encoding genetic algorithm (SEGA) is proposed for multitask-based anti-collision trajectory planning. Firstly, the spatial occupancy relationship between obstacles and manipulator is developed utilizing the theory of spherical enclosing box and spatial superposition. The obstacles are detected according to the relative position relationship between linear segments and spheres. Secondly, each joint trajectory between adjacent task areas is depicted with a sixth-degree polynomial. Additionally, each joint trajectory is improved via optimizing the unknown six-order coefficient. By searching for optimal sequential order of task areas, optimal collision detection results, and optimal joint trajectories, the multitask-based anti-collision trajectory planning problem is transformed into a parameter optimization problem. In SEGA, the cost function consists of two parts, including the end-effector path length and the variation of joint angles. Moreover, each chromosome consists of three categories of genes, including the sequential order of task areas, the sequential order of joint configurations corresponding to task areas, and the unknown coefficients for anti-collision joint trajectories. Finally, numerical simulations are carried out to verify the proposed method.

## 1. Introduction

With the fast development of science and technology, manipulator technology has been gradually applied in medical, aerospace, food inspection, and other fields. For complex industrial production, the redundant manipulator [1,2] provides great advantages. It offers high flexibility and offers strong obstacle avoidance ability by increasing the joint degrees of freedom. Moreover, the redundant manipulator can adapt to different working conditions and perform multiple tasks. The location and the size of obstacles vary in different working environments. The base of manipulator is fixed. Since the physical parameters of the manipulator are predefined, the manipulator workspace can be calculated. A large number of obstacles or large obstacles will lead to extremely limited manipulator workspace. In this case, the manipulator cannot successfully visit all the predefined multiple task points with any path. Without considering this extreme case, anti-collision trajectory planning [3,4] is the prerequisite for automated production lines utilizing manipulators, and the following aspects must be considered. The first aspect is the global anti-collision [5] of manipulator components, including the end-effector (EE) and the links. The second aspect denotes the smooth trajectories of manipulator components, including EE pose, EE velocity, joint angles, joint angular velocities, and joint angular accelerations. The third aspect depicts the safe and optimal anti-collision manipulator trajectories, with requirements of minimum time cost [6], efficient energy [7], etc.

Numerous studies focus on the anti-collision trajectory planning of manipulators. Qi [8,9] utilized genetic algorithm to search intermediate points for anti-collision trajectory planning of space manipulators. The intermediate points are employed for obstacle avoidance. The joint angular velocities and the angular accelerations are zero at each intermediate point. Dong et al. [10] calculates the collision-free path in a complex environment with multiple obstacles according to the density of manipulator workspace. Jiang et al. [11] studied the trajectory planning for a five-DOF (degree of freedom) manipulator utilizing the particle swarm optimization (PSO) [12,13] algorithm. Both EE path length and EE attitude variation are optimized. However, the PSO easily falls into the local optimum. Gai et al. [14] proposed an artificial potential field method for anti-collision trajectory planning of a six-DOF manipulator. The obstacles are formulated in joint space through constructed geometrical relationships. The obstacle repulsion vectors and the target point attraction vectors are designed for active anti-collision of links. In [15], each joint trajectory is broken up into several segments, where the intermediate points are optimized. The kinematic performance of manipulator is improved while achieving obstacle avoidance. However, the manipulator stops at intermediate points during movement, which cannot meet the practical application requirements.

Currently, two categories of approaches are employed for the study of multitask-based manipulator trajectory planning [16,17]. One is the decomposition solution method. The trajectory planning problem is decomposed into several subproblems [18], which are successively solved. The other one is the direct solution method [19]. Compared with decomposition methods, the direct method has advantages of higher accuracy, less computation, and shorter CPU time. Therefore, we use the direct solution method for multi-task trajectory planning and each task area is assumed to be a point. To improve the effectiveness and accuracy, numerous methods are developed for the inverse kinematics of a manipulator [20,21]. Zacharia et al. [20] assumed that each task point corresponds to two sets of manipulator configurations. The time consumption between adjacent task points is calculated. The optimal maneuvering time is achieved by optimizing the sequential order of task points and manipulator configurations. Baizid et al. [22,23] proposed to convert the multitask-based trajectory planning problem into an optimization problem. SEGAs are developed to optimize unknown parameters. A SEGA is also developed in [24], where each chromosome consists of three parts, including the sequential order of task points, the sequence of manipulator configurations, and intermediate manipulator configurations between adjacent task points. In addition, the obstacle avoidance factor was considered.

In this paper, the multitask-based anti-collision trajectory planning problem is studied, including the development of optimal sequential order of task points [25,26] and the study of optimal anti-collision joint trajectories between adjacent task points. Combined with an intelligent optimization algorithm, the trajectory planning problem is transformed into a parametric optimization problem with constraints. The manipulator is constrained to avoid obstacles. Additionally, joint angular velocities and angular accelerations are continuous.

The developed method is depicted as follows. First, the collision detection. Based on a simplified model, the collision detection calculation is carried out by judging the position relationship between each manipulator link and obstacles. Second, depiction of joint trajectories. Joint-space trajectory planning of the manipulator between task points via sixth-degree polynomials. The six-order coefficient is set as an adjustable parameter. By adjusting this parameter, the motion trajectory of the manipulator can be changed to bypass obstacles. Third, parameter optimization. The fitness function is established based on the collision detection results, the trajectory length of EE, and the rotation angle of each joint. The SEGA is used to optimize the sequential order of task areas, the sequential order of joint configurations corresponding to task areas, and the unknown coefficients for anti-collision joint trajectories. The optimal sequence of task points and the optimal adjustable parameters are obtained. In the joint space, an ideal multi-task and collision-free motion trajectory is planned. Moreover, the performance indexes of the trajectory are optimized, including the trajectory length and rotation angle.

The rest of this paper is organized as follows. Section 2 introduces the model simplification, including the model of the redundant manipulator, and the occupying relationship between the manipulator and the obstacle. The collision detection scheme is introduced in Section 3. In Section 4, the joint trajectories are depicted between adjacent task points. The IGA encoding and updating mechanisms, together with the SEGA optimization process, are introduced in Section 5. In Section 6, numerical simulations are carried out to validate IGA, including comparisons with other approaches. Section 7 concludes the work.

## 2. Model Simplification

### 2.1. Redundant Manipulator Model

**Figure 1:**
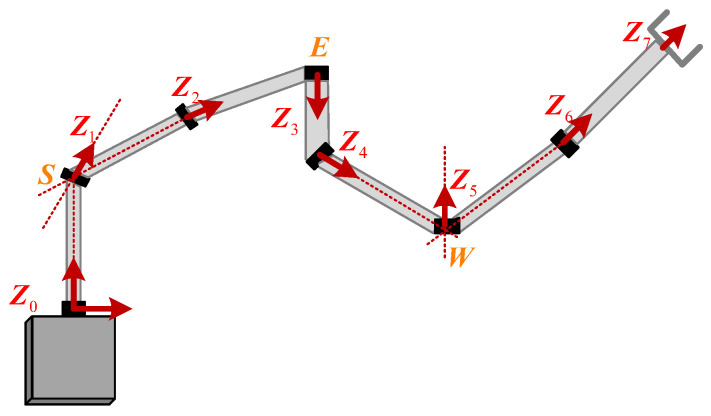
Joint layout of S-R-S manipulator.

**Figure 2:**
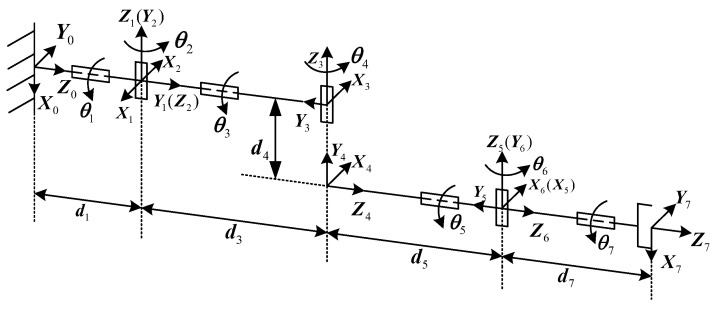
D-H frames of S-R-S manipulator.

Figure 1 shows the joint layout of the developed redundant manipulator, which is a spherical–revolute–spherical (S-R-S) [27,28] manipulator. The S-R-S manipulator is similar to a human arm and is often used to perform dexterous tasks.The shoulder joints of the S-R-S manipulator denote joint 1, joint 2, and joint 3. The elbow joint denotes joint 4. The wrist joints denote joint 5, joint 6, and joint 7. According to the characteristics of the S-R-S manipulator, the shoulder joints intersect at one point denoted as *S*, the elbow joint is denoted as *E*, while the wrist joints intersect at one point denoted as *W*. Figure 2 shows the established Denavit–Hartenberg(D-H) coordinate systems of the S-R-S manipulator, and the D-H parameters are listed in Table 1.

The S-R-S manipulator has seven DOFs and is capable of performing complicated tasks owing to its adaptability and flexibility. The kinematic inverse [29,30] solutions of the S-R-S manipulator is infinite due to the redundancy of the DOFs. A task point in the operation space corresponds to infinite joint configurations in the joint space. For each task point, the corresponding joint configurations are studied. Because of the singularity of the single-arm angle parametrization, the concept of dual arm-angle parametrization [31,32] is introduced to optimize joint configurations. Finally, eight sets of joint configurations are obtained, which are corresponding to one task point in the operation space.

### 2.2. Space Occupying Relationship Between Manipulators and Obstacle

**Figure 3:**
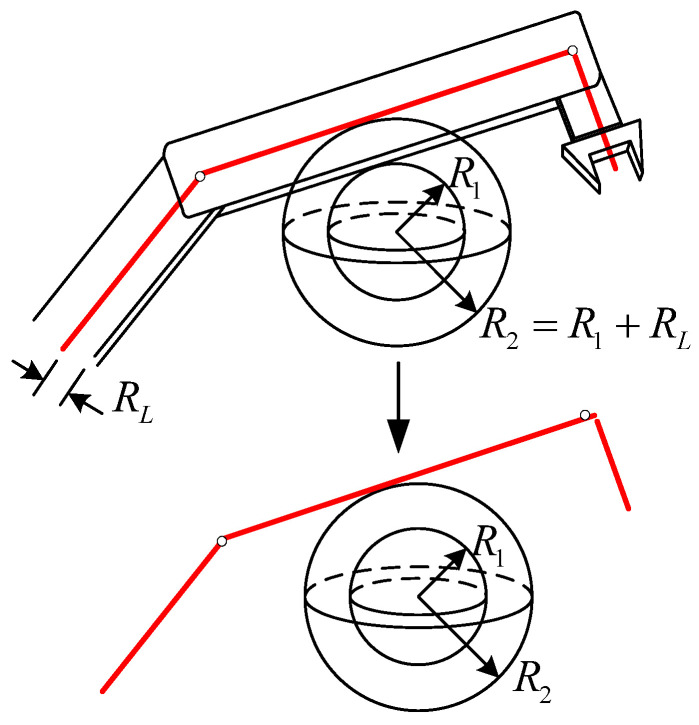
Simplification of manipulator links.

Most manipulator links are rectangular or cylindrical rigid bodies, making collision detection between manipulators and obstacles very complicated. In order to facilitate the calculation, the concept of the spherical enveloping box is introduced and the method of spatial superposition is developed to simplify the manipulator links and obstacles. The radial maximum spherical envelope box radius of the manipulator link is superimposed on the thickness of the obstacle. Thus, the spatial occupancy relationship between the manipulator and obstacle is turned into the relative position relationship between the linear segment and the sphere, as shown in Figure 3.

An obstacle enclosing sphere is defined as the smallest sphere that can completely enclose an obstacle. The line between the two farthest points on the obstacle is generally chosen as the diameter of enclosing sphere, formulated as:

(1) $$R={\left(x,y,z\right)|\left(x-a\right)}^{2}+{\left(y-b\right)}^{2}+{\left(z-c\right)}^{2}\leq r^{2}$$

where (a,b,c) is the center coordinate of enclosing sphere, *r* is the radius of enclosing sphere, (x,y,z) denotes the coordinate of any point on the obstacle surface. This method is intuitive and is easy to calculate, reducing the difficulty of follow-up collision detection.

## 3. Collision Detection

Based on the simplified model in Section 2, the collision detection is studied between the manipulator and obstacles. The relative positional relationship is established between each link and each obstacle, where each link is simplified as a segment and each obstacle is depicted as an enclosing ball.

**Figure 4:**
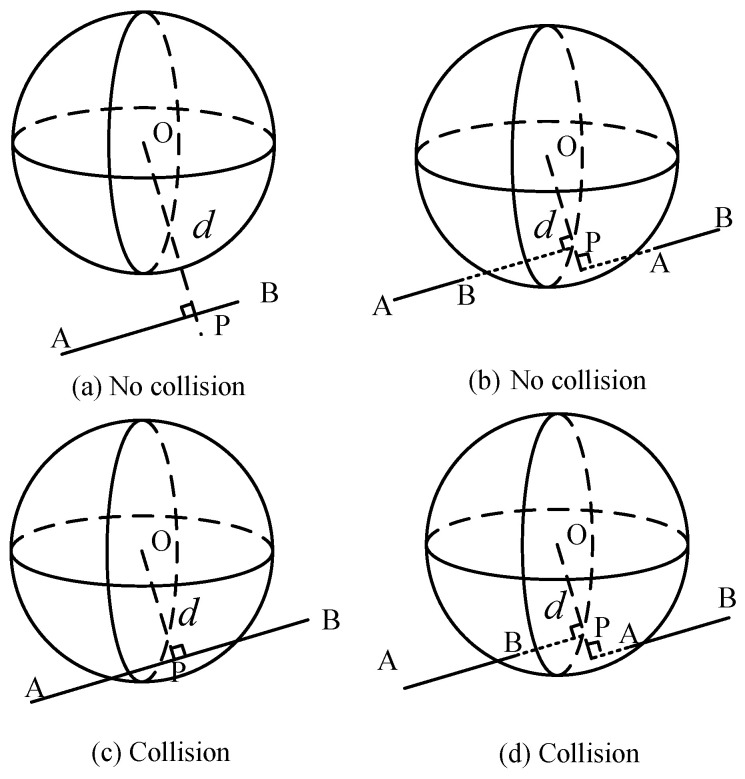
Collision detection results between a manipulator link and an obstacle, with (**a**) no collision, (**b**) no collision, (**c**) collision with 2 intersection points, and (**d**) collision with 1 intersection point.

Figure 4 represents the positional relationship between a link and an obstacle, where the link is depicted as a line segment AB and the obstacle is a sphere with center *O*. The radius of the sphere is defined as *r*. The distance between the point *O* and the segment AB is denoted as *d*, with the foot point *P*. Then, the following formulations are established.

(2) $$t=\vec{AO}\cdot \vec{AB}/\left|\right|\vec{AB}{\left|\right|}^{2}$$

(3) $$d=|\vec{OP}\left|\right|=||t\vec{AB}+\vec{AO}\left|\right|$$

(4) $$\vec{AB}\cdot \left(t*\vec{AB}+\vec{AO}\right)=0$$

where $\vec{AB}$, $\vec{AO}$, and $\vec{OP}$ are three vecotrs, and ||·|| denotes the module value of a vector. The collision detection results, between a manipulator link and a spherical obstacle, can be categorized into four cases.

(1) d>r, the link does not collide with the obstacle, as shown in Figure 4a.
(2) d<r, the point *P* is on the extended line of segment AB. The values of *t* in (2) are categorized as follows. First, t<0, $\left(|\right|\vec{AB}{\left||*|t|\right)}^{2}+d^{2}>r^{2}$. Second, t>1, $\left(|\right|\vec{AB}||*|t-{1|)}^{2}+d^{2}>r^{2}$. In this case, the segment AB does not intersect the sphere. There is no collision, as shown in Figure 4b.
(3) d≤r, the segment AB intersects with the sphere at two points, where 0<t<1. In this case, the link collides with the obstacle, as shown in Figure 4c.
(4) d<r, the point *P* lies on the extended line of segment AB. The segment AB interacts with the sphere at one point. The values of *t* in (2) are categorized as follows. First, t<0, $\left(|\right|\vec{AB}{\left||*|t|\right)}^{2}+d^{2}\leq r^{2}$. Second, t>1, $\left(|\right|\vec{AB}||*|t-{1|)}^{2}+d^{2}\leq r^{2}$. In this case, the manipulator link collides with the obstacle, as shown in Figure 4d.

Based on the relative position between each link and obstacles, the collision result is judged between the manipulator and obstacles at a certain time. An iterative detection strategy is employed for collision detection during the whole trajectory planning process. The trajectory is divided into a series of interpolation points. For each interpolation point, the collision result is judged between the manipulation and obstacles. By traversing the trajectory sequence, a series of Boolean values are calculated about collision results. Therefore, the collisions for the whole trajectory are detected.

## 4. Depiction of Joint Trajectories Between Adjacent Task Points

The manipulator trajectory is planned in joint space. The fifth-degree polynomial [33,34] is usually employed for depiction of joint trajectories to guarantee the smooth joint angular acceleration and to avoid the generation of shocks and vibrations. With this case of depiction, only one manipulator movement solution is derived. Because of the single solution, the manipulator usually collides with obstacles. In addition to producing smooth and continuous trajectories, a sixth-degree polynomial has enough degrees of freedom to satisfy various constraints. It can increase a coefficient and adjust the trajectory by changing the coefficient, so as to achieve the purpose of obstacle avoidance. At the same time, compared with higher degree polynomials, the sixth-degree polynomial has relatively low computational complexity while maintaining enough smoothness and flexibility. It can meet the real-time requirements while ensuring the accuracy. Therefore, each joint trajectory between adjacent task points is depicted with a sixth-degree polynomial. Additionally, the six-order coefficient is set as an adjustable parameter. By adjusting this parameter, the motion trajectory of the manipulator can be changed to bypass obstacles. The sixth-degree polynomial is formulated as:

(5) $$\theta \left(t\right)=a_{0}+a_{1}t+a_{2}t^{2}+a_{3}t^{3}+a_{4}t^{4}+a_{5}t^{5}+Kt^{6}$$

where *K* is the adjustable coefficient. $\theta =[\theta_{1},\theta_{2},\theta_{3},\theta_{4},\theta_{5},\theta_{6},\theta_{7}]$, and where $\theta_{i}\;\left(i=1,\ldots ,7\right)$ denotes the *i*th joint angle. $K=[K_{1},K_{2},K_{3},K_{4},K_{5},K_{6},K_{7}]$, where $K_{i}$ denotes the highest-degree coefficient of polynomial $\theta_{i}\left(t\right)$. According to the derivation of (5), the joint angular velocity is formulated as:

(6) $$\dot{\theta (t)}=a_{1}+2a_{2}t+3a_{3}t^{2}+4a_{4}t^{3}+5a_{5}t^{4}+6Kt^{5}$$

The joint angular acceleration is formulated as:

(7) $$\ddot{\theta }\left(t\right)=2a_{2}+6a_{3}t+12a_{4}t^{2}+20a_{5}t^{3}+30Kt^{4}$$

At the initial moment $t_{0}$, the joint angle, the angular velocity and the angular acceleration are, respectively, depicted as $\theta_{t_{0}}$, ${\dot{\theta }}_{t_{0}}$, and ${\ddot{\theta }}_{t_{0}}$. At the final moment $t_{f}$, the joint angle, the angular velocity, and the angular acceleration are, respectively, depicted as $\theta_{t_{f}}$, ${\dot{\theta }}_{t_{f}}$, ${\ddot{\theta }}_{t_{f}}$. To ensure smooth joint trajectories without abrupt changes, the angular velocity and the angular acceleration are constrained to be zero at initial and final moments. Therefore, six boundary conditions are derived as:

(8) $$\left\{\begin{array}{cc} \theta (t_{0}) & =a_{0}\\ \dot{\theta }\left(t_{0}\right) & =a_{1}\\ \ddot{\theta }\left(t_{0}\right) & =a_{2}\\ \theta (t_{f}) & =a_{0}+a_{1}t_{f}+a_{2}t_{f}^{2}+a_{3}t_{f}^{3}+a_{4}t_{f}^{4}+a_{5}t_{f}^{5}+Kt_{f}^{6}\\ \dot{\theta }\left(t_{f}\right) & =a_{1}+2a_{2}t_{f}+3a_{3}t_{f}^{2}+4a_{4}t_{f}^{3}+5a_{5}t_{f}^{4}+6Kt_{f}^{5}\\ \ddot{\theta }\left(t_{f}\right) & =2a_{2}+6a_{3}t_{f}+12a_{4}t_{f}^{2}+20a_{5}t_{f}^{3}+30Kt_{f}^{4} \end{array}\right.$$

In addition, the coefficients are formulated as:

(9) $$\left\{\begin{array}{cc} a_{0} & =\theta_{0}\\ a_{1} & =\omega_{0}\\ a_{2} & =\frac{1}{2}\alpha_{0},\\ a_{3} & =\frac{20\left(\theta_{f}-\theta_{0}\right)-\left(8\omega_{f}+\omega_{0}\right)t_{f}-\left(3\alpha_{0}-\alpha_{f}\right)t_{f}^{2}-2Kt_{f}^{6}}{2t_{f}^{3}}\\ a_{4} & =\frac{15\left(\theta_{0}-\theta_{f}\right)+\left(7\omega_{f}+8\omega_{0}\right)t_{f}+\left(0.5\alpha_{0}-\alpha_{f}\right)t_{f}^{2}+3Kt_{f}^{6}}{2t_{f}^{4}}\\ a_{5} & =\left(\frac{12\left(\theta_{f}-\theta_{0}\right)-\left(6\omega_{f}+6\omega_{0}\right)t_{f}-\left(\alpha_{0}-\alpha_{f}\right)t_{f}^{2}-6Kt_{f}^{6}}{2t_{f}^{5}}\right) \end{array}\right.$$

where θ, ω, α denote the joint angle, angular velocity, and angular acceleration, respectively. If the optimal value of *K* is determined, the values of coefficients $a_{3}$, $a_{4}$, and $a_{5}$ are calculated through (9). Therefore, the joint trajectory between adjacent task points is derived.

## 5. Special Encoding Genetic Algorithms

The genetic algorithm (GA) belongs to evolutionary algorithms, contributing to searching for optimal solutions by imitating the mechanism of selection and inheritance in nature. First, individuals are selected for population initialization. Then, the fitness values of individuals are calculated, where individuals with adequate fitness values are selected as parents through methods such as roulette. Finally, the operations of crossover and mutation are successively performed to produce offspring.

Three factors are considered during the multitask-based anti-collision trajectory planning of redundant manipulators, including the optimal sequential order of task points, the optimal sequential order of joint configurations, and the optimal unknown coefficients of sixth-degree polynomials. Therefore, three types of genes are studied. Since GA only optimizes one type of gene, GA is executed three times. The sequence of task points is obtained first. Second, the sequence of joint configurations is optimized. Third, the joint trajectories between adjacent task points are optimized. The accuracy of the final result is firstly reduced due to the division mechanism. Secondly, both the calculation and the CPU time increase because of the multiple times of running the traditional GA. The SEGA is an effective method to determine the optimal path sequence of the EE to multiple task points when considering multiple solutions of inverse kinematics problem. It can quickly find the optimal or near-optimal solution in an affordable time and reduce computation and CPU time. At the same time, the multiple configurations of arbitrary non-redundant operators can be easily reflected in the genetic algorithm coding, considering the global optimization of the problem and finally improving the optimization efficiency. Inspired by the literatures [16,22], an SEGA is developed to simultaneously optimize three different types of genes, including the sequence of task points, the sequence of joint configurations corresponding to the sequence of task points, and unknown sixth-degree polynomial coefficients. The three parts are separately encoded. In addition, the crossover and the mutation mechanisms are independently designed.

### 5.1. Cost Function

During manipulator performance of multiple tasks, high efficiency is required to reduce unnecessary movements while meeting anti-collision constraints. Therefore, the optimal EE path length and the joint angular variations are studied. The cost function consists of three components, including the EE path length $f_{L}$, the angular variation of each joint $f_{Q}$, and the collision detection result $f_{CO}$. $f_{L}$ and $f_{Q}$ are formulated as:

(10) $$f_{Q}=\sum_{i=1}^{n}\sum_{i=1}^{n}\left(\right|\theta_{i+1,j}-\theta_{i,j}\left|\right)$$

(11) $$f_{L}=\sum_{i=1}^{n}\sqrt{{\left(p_{i+1,x}-p_{i,x}\right)}^{2}+{\left(p_{i+1,y}-p_{i,y}\right)}^{2}+{\left(p_{i+1,z}-p_{i,z}\right)}^{2}}$$

where *p*, θ denote the coordinates of the actuator at the end of the robot arm and the joint angle, respectively. The cost function $f_{K}$ is the combination of $f_{Q}$, $f_{L}$, $f_{CO}$, shown as:

(12) $$f_{K}=-\frac{f_{CO}}{\eta_{1}f_{Q}+\eta_{2}f_{L}}$$

In (12), $f_{CO}$ denotes the result of collision detection. If the manipulator has no collision with obstacles, we have $f_{CO}=1,f_{K}<0$. Conversely, if a collision occurs, we have $f_{CO}=0,\;f_{K}=0$. Since the fitness values without collisions are always less than values with collisions, infeasible individuals are directly screened out during optimization. The scales of $f_{Q}$ and $f_{L}$ are dissimilar, and the orders of magnitudes are different. In order to ensure that $f_{Q}$, $f_{L}$ share the same order of magnitude, the weighting factors $\eta_{1}$ and $\eta_{2}$ are considered in (12).

### 5.2. Chromosome Encoding Mechanism

**Figure 5:**
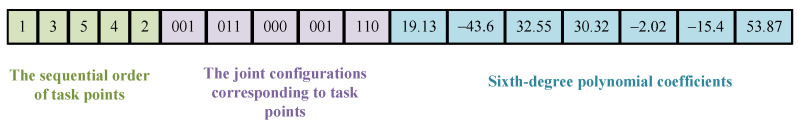
Encoding mechanisms of one chromosome in SEGA.

In the SEGA, each chromosome contains three components, i.e., the sequential order of task points, the sequence of joint configurations, and the unknown sixth-degree polynomial coefficients. Figure 5 shows the encoding mechanism of three components for each chromosome.

A. Encoding for the first part

In Figure 5, the first part represents the sequential order of task points. Each task point is visited by the end effector of the manipulator and is limited to being visited once. Integer encoding is the best choice. For the case with *N* task points, the first part of each chromosome consists of *N* genes. Each gene is assigned with a value among *N* numbers from 1 to N−1 and is not the same with other N−1 genes.

B. Encoding for the second part

**Figure 6:**
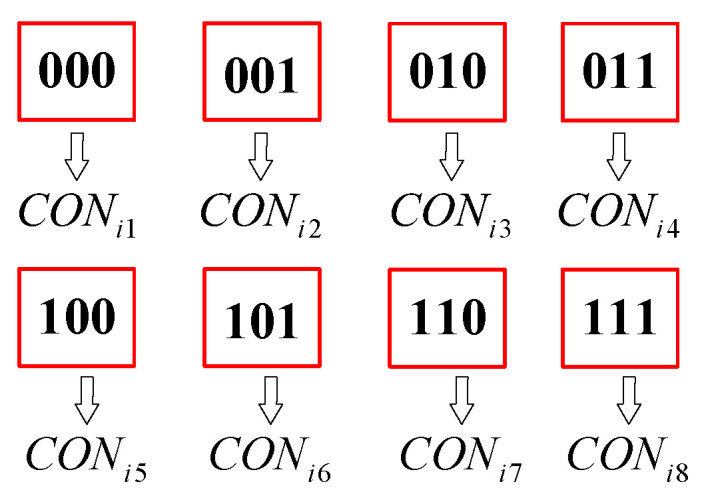
A binary representation of eight robotic arm configurations.

In Figure 5, the second part represents the sequence of joint configurations corresponding to the sequential order of task points. For each task point, eight pairs of joint configurations are obtained. Therefore, each component of the second part has eight choices and the values of components are consistent after decoding. In this second part, the binary encoding mechanism is selected. Since eight special joint configurations corresponding to one waypoint are considered, each joint configuration is coded by one byte of three bits. The first set of manipulation configurations is represented by the byte “000”, the second configuration is represented by the byte “001”, and so on. Finally, the eighth configuration is represented by the byte “111”. Figure 6 represents eight sets of joint configurations corresponding to one task point, where $CON_{im}\;\left(i=1,\ldots ,n;m=1,\ldots ,8\right)$ denotes the *m*th joint configuration at task point *i* and is expressed as:

(13) $$CON=\left[\begin{array}{cccc} CON_{11} & CON_{12} & \ldots & CON_{18}\\ CON_{21} & CON_{22} & \ldots & CON_{28}\\ \ldots & \ldots & \ldots & \ldots \\ CON_{n1} & CON_{n2} & \ldots & CON_{n8} \end{array}\right]$$

During the end-effector of the manipulator moving from the *i*th task point to the (i+1)th point, $CON_{im}\;\left(m=1,\ldots ,8\right)$ denotes the joint configuration of $\theta =[\theta_{1},\theta_{2},\theta_{3},\theta_{4},\theta_{5},\theta_{6},\theta_{7}]$, relative to task point i, at initial time $t_{0}$ in Equations (5) and (8). $CON_{i+1,m},m\;\left(m=1,\ldots ,8\right)$ denotes the joint configuration of $\theta =[\theta_{1},\theta_{2},\theta_{3},\theta_{4},\theta_{5},\theta_{6},\theta_{7}]$, relative to task point i+1, at final time $t_{f}$ in Equations (5) and (8).

C. Encoding for the third part

In Figure 5, the second part represents unknown sixth-degree polynomial coefficient *K*, where $k=[K_{1},K_{2},K_{3},K_{4},K_{5},K_{6},K_{7}]$. Large and small values of *K* lead to instantaneous variation of trajectory curves, resulting in the over-limit of joint torque. Therefore, the value of *K* is constrained to be within the region of [−500,500]. Furthermore, the real number encoding mechanism is selected in this third part.

### 5.3. Bio-Inspired Genetic Mechanism

After population initialization, the individuals are repetitively updated through bio-inspired genetic mechanisms, including the selection mechanism, the crossover mechanism, and the mutation mechanism.

#### 5.3.1. Chromosome Selection Mechanism

A predefined number of individuals are selected at the initial phase of each generation. The roulette wheel mechanism is employed for the selection. The individuals with high-quality solutions are preferentially selected. In this work, the high-quality solutions denote the individuals with comparatively small fitness values.

#### 5.3.2. Chromosome Crossover Mechanism

After the selection process, three crossover mechanisms are respectively employed for each part of chromosomes.

A. Crossover for the first part

**Figure 7:**
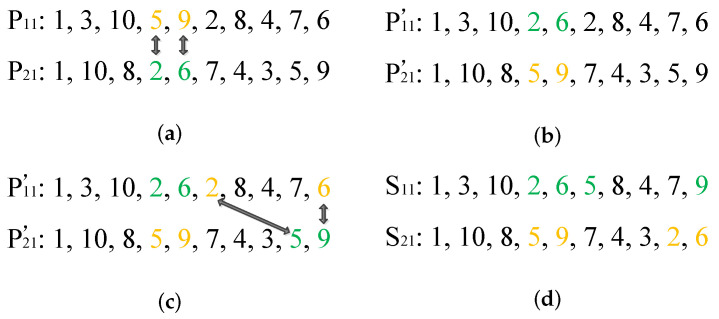
PMX operation for the first part of the chromosome: (**a**) parents, (**b**) transitional chromosomes, (**c**) transitional chromosomes with mapping, and (**d**) sons.

The partially matched crossover (PMX) mechanism is utilized for the first part of the chromosomes. Figure 7 presents an example of PMX, where $P_{11}$ and $P_{21}$ are two parents, and $S_{11}$ and $S_{21}$ are two sons. In Figure 7a, two genes of each parent are selected, and the selected genes are in the same position of two parents. In Figure 7b, the genes in the same position are exchanged, where the transitional chromosomes are denoted as $P_{11}^{\prime }$ and $P_{21}^{\prime }$, respectively. In Figure 7c, the repeated genes are marked and will be exchanged by mapping {2} to {5} and {6} to {9}, represented by a two-way arrow in Figure 7c. After exchanging genes, the sons are generated as shown in Figure 7d.

B. Crossover for the second part

**Figure 8:**
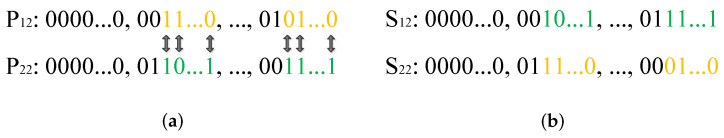
One-point crossover operation for the second part of the chromosome: (**a**) parents, and (**b**) sons.

The second part of the chromosome denotes the sequence of joint configurations. The segmented one-point crossover mechanism is utilized in this part. Figure 8 presents an example of one-point crossover. For each sub-part of parents $P_{12}$ and $P_{22}$, the cut points are first randomly selected in the same position of $P_{12}$ and $P_{22}$. Then, from the cut point to the last point, the genes of $P_{12}$ and $P_{22}$ are exchanged by mappings, as shown in Figure 8a. The generated sons $S_{12}$ and $S_{22}$ are shown in Figure 8b.

C. Crossover for the third part

**Figure 9:**
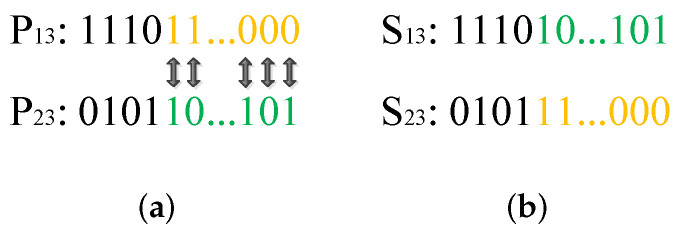
One-point crossover operation for the third part of the chromosome: (**a**) parents, and (**b**) sons.

Similar to the second part of the chromosome, the one-point crossover mechanism is utilized for the third part. An example is shown in Figure 9. The cut points of parents $P_{13}$ and $P_{23}$ are firstly selected. Then, the marked genes of parents are exchanged as shown in Figure 9a. The sons $S_{13}$ and $S_{23}$ are finally generated, as shown in Figure 9b.

#### 5.3.3. Chromosome Mutation Mechanism

The mutation operation follows the crossover operation and is independently performed for the three parts of chromosomes. Inspired by [20], the mutation rate is set to be within the region of [0.1,0.4]. Additionally, the three parts share the same mutation rate.

A. Mutation for the first part

**Figure 10:**
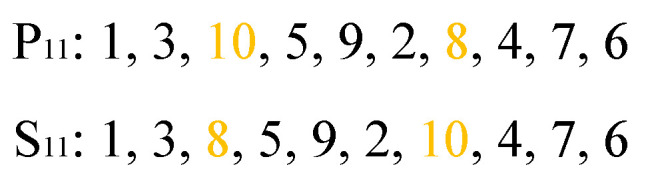
Mutation operation for the first part of the chromosome.

Regarding the mutation operation for the first part of chromosome, two genes are randomly selected and exchanged with each other, as shown in Figure 10. In Figure 10, $P_{11}$ and $S_{11}$ are, respectively, the parent and the son.

B. Mutation for the second part

**Figure 11:**
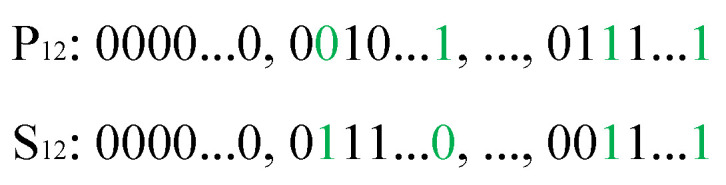
Mutation operation for the second part of the chromosome.

The segmented mutation mechanism is employed for the second part of the chromosome. In each sub-part of parent $P_{12}$, two genes are randomly selected and each value is changed from 1 to 0 and vice versa, as shown in Figure 11.

C. Mutation for the third part

**Figure 12:**
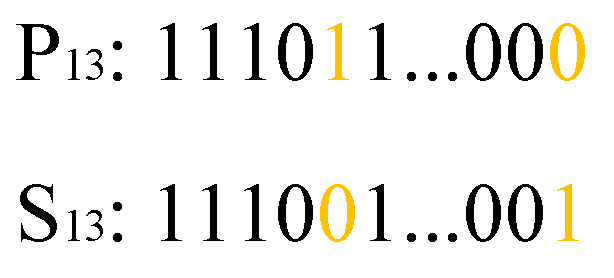
Mutation operation for the third part of the chromosome.

Regarding the mutation operation for the third part of the chromosome, two genes are randomly selected. The value of each gene is changed from 0 to 1 and vice versa, as shown in Figure 12.

### 5.4. Special Encoding Genetic Algorithm Optimization Process

**Figure 13:**
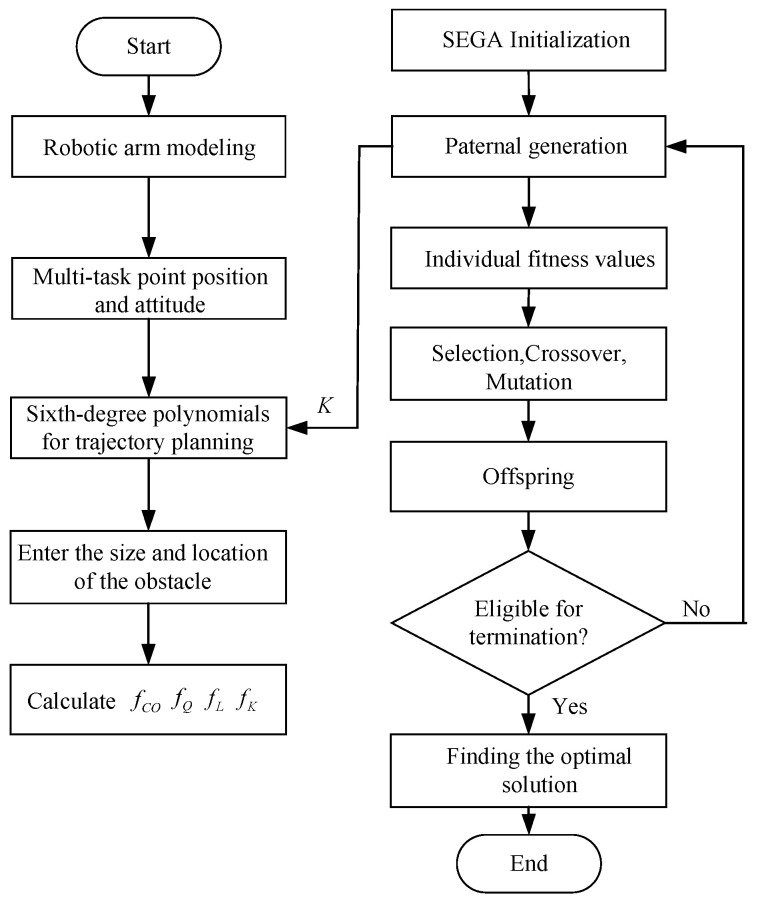
Special encoding genetic algorithm flow.

The overall flow of the SEGA is shown in Figure 13. First, the obstacles and the manipulator are modelled, together with the initialization of individuals. The population size of SEGA is *M*. *M* solutions are generated during SEGA initialization. The fitness values of $f_{K}$ in (12) are calculated by combing the collision detection result $f_{CO}$, the total amount of angular variation of each joint $f_{Q}$, and the end-effector trajectory length $f_{L}$. While $f_{K}=0$, the related solution is omitted. The omitted solutions will be replaced by newly generated solutions at the beginning of next iteration, so that the population size of the SEGA is *M* in each iteration. Then, the selection operation is performed utilizing a roulette mechanism. After this, the crossover and the mutation operations are, respectively, performed by each part of the chromosomes to obtain the offspring. The termination condition is finally checked to find the optimal individual that minimizes the cost function.

## 6. Numerical Simulations

In order to verify the effectiveness of the SEGA, numerical experiments are carried out under the MATLAB environment. The seven-DOF S-R-S manipulator is constrained to execute multiple tasks in travel considering different numbers of obstacles. The S-R-S manipulator is modeled utilizing MATLAB Robotics Toolbox. The maximum radial radius of manipulator links is set as $R_{L}=0.03$ m. Additionally, boundary balls are used to simulate obstacles. In the SEGA, the roulette wheel mechanism is employed for the selection process. The population size is set to be 200, and the number of iterations is 200. The crossover and the mutation probabilities are defined as 0.6 and 0.15, respectively.

### 6.1. Comparisons

#### 6.1.1. Verification of Anti-Collision Algorithm SEGA

Two cases of simulations are developed in this part, with and without obstacles during manipulator movement. The position and attitude of five task points are listed in Table 2. The coordinates of the obstacle center are set as O=[1.0,0.8,0.5], and the obstacle radius is set as $R_{1}=0.15$ m. Therefore, the safe distance is calculated as $S=R_{1}+R_{L}=0.18$ m. In addition, the motion time of manipulator is set as 4 s. The number of interpolation points is set as 80, i.e., the running time between each segment of interpolation points is 0.05 s.

**Figure 14:**
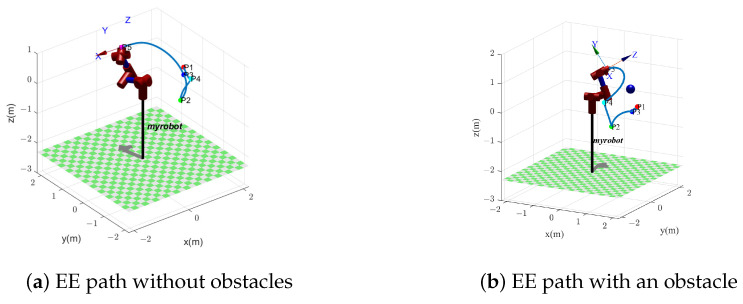
The manipulator EE path in two cases: (**a**) without obstacle; (**b**) with an obstacle.

Figure 14 shows the motion path of manipulator EE when performing five tasks, where each task area is assumed to be a point. The pose of five points is shown in Table 2. Figure 14a shows EE path without obstacles, while Figure 14b shows EE path with an obstacle. In both cases, the EE visits each task point once and only once. However, because of the obstacle, the manipulator movements in Figure 14b differ from the movements in Figure 14a. In Figure 14a, the sequential order of visited task points is $P_{1}\to P_{4}\to P_{2}\to P_{3}\to P_{5}$. In Figure 14b, an obstacle is considered, and the EE passes through the task points in the order of $P_{1}\to P_{3}\to P_{2}\to P_{4}\to P_{5}$.

#### 6.1.2. Comparison Between GA and SEGA

In order to validate the effectiveness of the SEGA, comparisons are developed between GA and SEGA. The multitask-based anti-collision trajectory planning utilizing GA is divided into two sub-problems which are successively solved. First, the sequential order of task points is obtained utilizing GA. Based on the optimal sequential order, anti-collision joint trajectories are then planned between adjacent task points,. In summary, the GA runs *N* times, where *N* denotes the number of task points. However, the SEGA runs once. In addition, in order to compare the two algorithms, the parameter settings should be unified.

**Figure 15:**
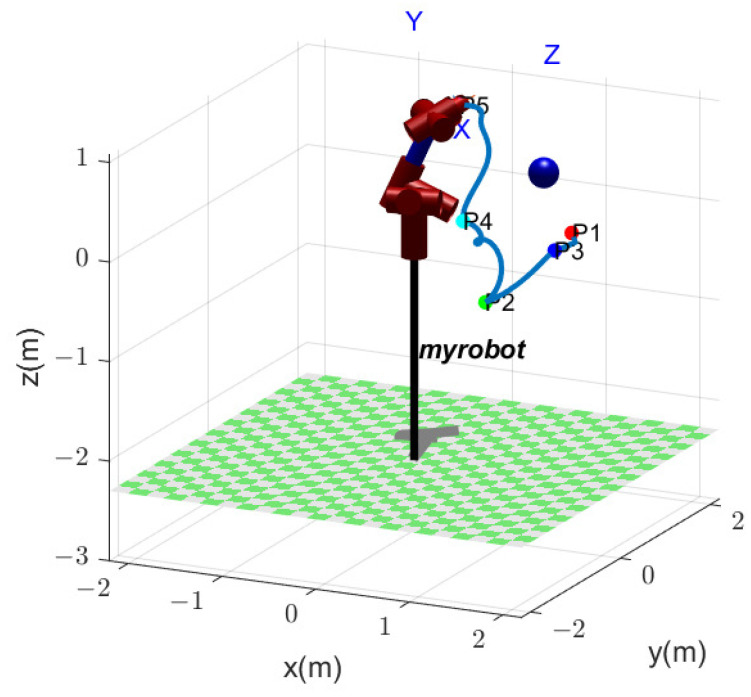
Anti-collision EE path utilizing GA.

**Figure 16:**
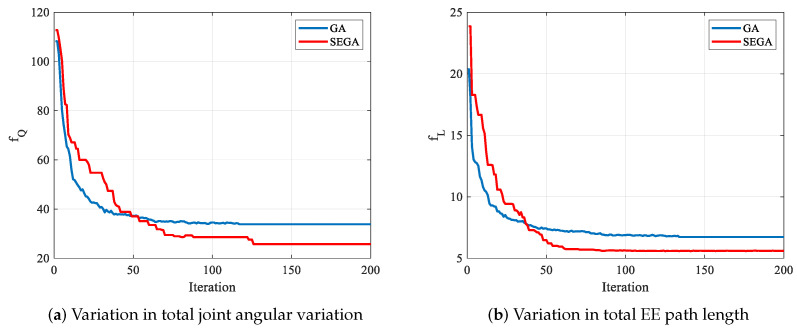
Comparisons between GA and SEGA, where (**a**) variation in total joint angular variation; (**b**) variation in total EE path length.

Figure 15 shows EE path utilizing the GA. The manipulator components have no collision with the obstacle. The EE passes through the task points in the order of $P_{1}\to P_{3}\to P_{2}\to P_{4}\to P_{5}$. Figure 16 shows the comparison results, including the total joint angular variation in Figure 16a and the EE path length in Figure 16b. The optimal joint angular variation is $f_{Q}=33.8356$ rad utilizing the GA, while the optimal value is $f_{Q}=25.7416$ rad utilizing the SEGA. As shown in Figure 16a, the optimal value is derived atthe 135th iteration utilizing the GA and at the 127th iteration utilizing the SEGA. The optimal EE path length is $f_{L}=6.73057$ m utilizing the GA, while the optimal value is $f_{L}=5.59401$ m utilizing the SEGA. As shown in Figure 16b, the optimal value is derived at the 134th iteration utilizing the GA and at 112th iteration utilizing the SEGA.

In the above comparison experiment, the GA runs four times. The unknown coefficients are also optimized four times. The computational complexity of utilizing the GA is four times of that utilizing the SEGA, where the unknown coefficients are optimized once with the SEGA. The CPU time is 1071 s utilizing the GA, while the CPU time is 859 s utilizing the SEGA. Therefore, compared with the GA, the SEGA has the advantages of less computation, higher precision, and less CPU consumption.

### 6.2. Manipulator Components Motions with Variable Obstacles

In this part, the SEGA is further validated via considering variable obstacles. Two cases are studied for manipulator component trajectories. One obstacle is considered in case 1, while two obstacles are considered in case 2. The number of task points is five in both cases, and case 1 is shown in Table 2. Compared with case 1, the third task point $P_{3}$ in case 2 is changed, where the position is [0.60 m; −0.50 m; 1.00 m] and the attitude is [0; 80; 80] (deg).

#### 6.2.1. Case 1: One Obstacle

**Figure 17:**
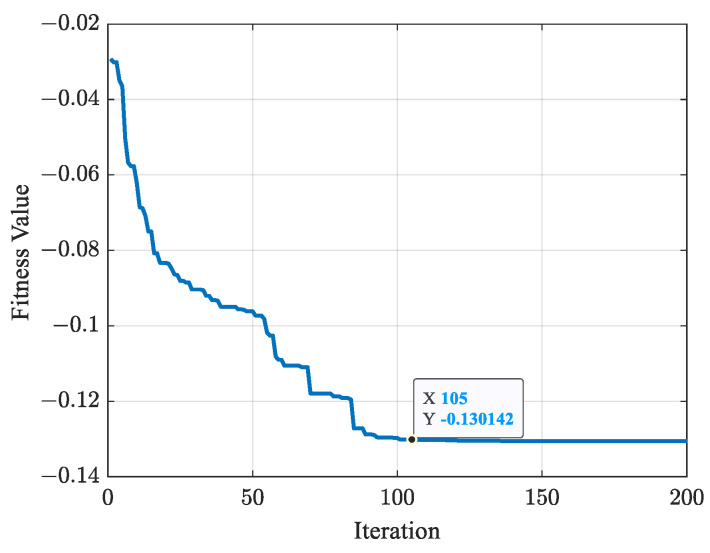
Variation curve of fitness values.

Figure 17 shows the variation in fitness values of cost function $f_{K}$ in (12). After 105 iterations, the optimal fitness value is obtained as $f_{K}=-0.130142$, with the optimal parameter K=[9.46,−6.01,1.47,11.51,0.03,−11.31,−0.98]. The total joint angular variation is $f_{Q}=22.5055$ rad, and the total EE path length is $f_{L}=5.4104$ m.

The EE path is shown in Figure 14b, where the EE passes through the task points in the order of $P_{1}\to P_{3}\to P_{2}\to P_{4}\to P_{5}$.

**Figure 18:**
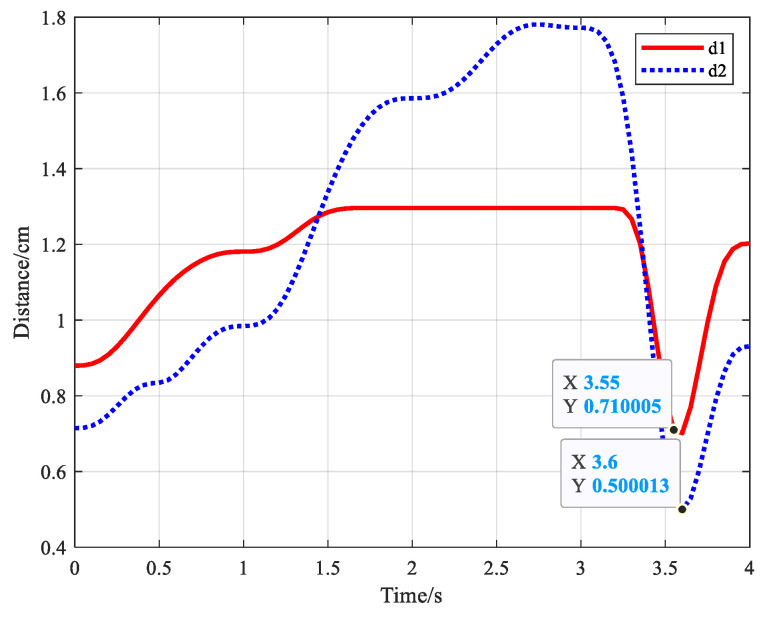
Distance variation between simplified S-R-S links and obstacle.

According to the D-H parameters in Table 1, the S-R-S manipulator can be approximated and simplified into a two-link mechanical arm. Figure 18 shows the variation in distance between obstacle center and simplified link i, where i = 1,2. The minimum distance between simplified link 1 and the obstacle is $d_{1}=0.710005$ m > 0.18 m, while the minimum distance between simplified link 2 and the obstacle is $d_{2}=0.500013$ m > 0.18 m. Therefore, the minimum distance between simplified S-R-S manipulator and obstacle is larger than the safety distance. In conclusion, the manipulator has no collision with the obstacles during movements.

**Figure 19:**
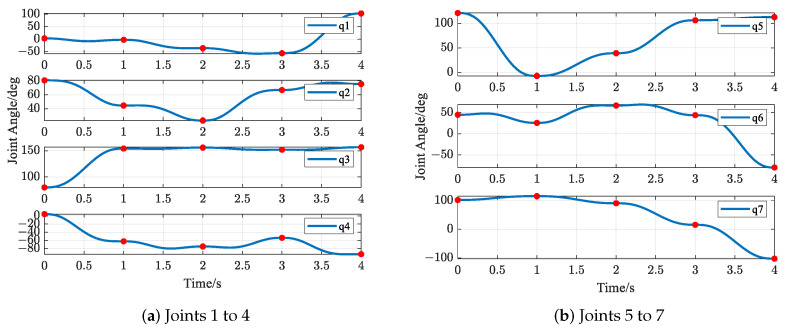
Profiles of angles in joint space.

**Figure 20:**
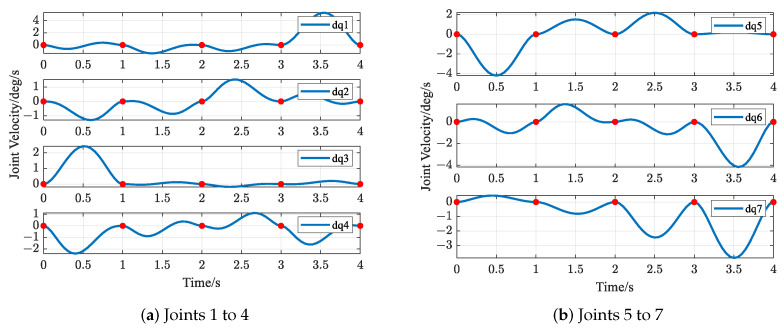
Profiles of angular velocity in joint space.

**Figure 21:**
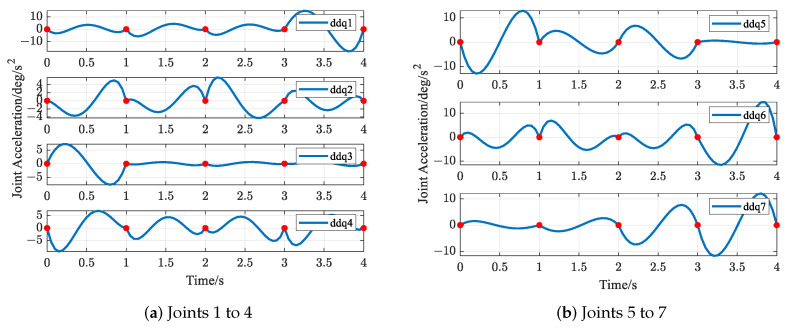
Profiles of angular acceleration in joint space.

**Figure 22:**
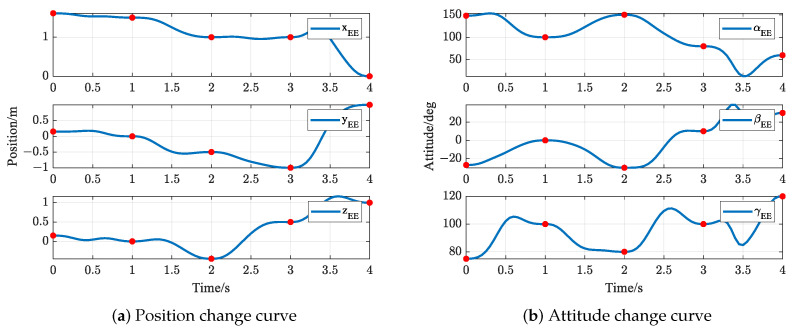
End-effector trajectory.

The trajectories of S-R-S components are shown in Figure 19, Figure 20, Figure 21 and Figure 22, including the joints and the EE. The red solid points in the figures indicate the corresponding values at the task points. Figure 22 illustrates the trajectory of the end-effector. In Figure 22b, $\alpha_{EE}$, $\beta_{EE}$ and $\gamma_{EE}$ denote the Euler angles around the $X_{EE}$ axis, $Y_{EE}$ axis, and $Z_{EE}$ axis, respectively. In Figure 22, the end-effector reaches each task point exactly once and only once and passes through the task points in the order of $P_{1}\to P_{3}\to P_{2}\to P_{4}\to P_{5}$. The variations in joint angles, annular velocities, and angular accelerations are, respectively, shown in Figure 19, Figure 20 and Figure 21. As shown in Figure 19, the manipulator does not have violent movement, each joint angular curve is smooth, and each joint angle is within the constraint range [−180,180] (deg). In Figure 20 and Figure 21, each joint angular velocity and each joint angular acceleration are zero at each task point. Therefore, the multitask-based anti-collision optimal trajectory of the S-R-S manipulator is derived, where an obstacle is considered.

The simulation results, including the joint trajectories, the joint angular trajectories, and the joint angular acceleration trajectories, are actually reference trajectories during performing real-world experiments. Combing the simulation results and the positive dynamics of the manipulator, the force acting on each joint of the manipulator is derived. It provides stronger evidence for subsequent physical or real-world experiments.

#### 6.2.2. Case 2: Two Obstacles

Other conditions remain unchanged; two obstacles are set up in the space. For obstacle 1, the center coordinate is set as $O_{1}=\left[1.0,0.8,0.5\right]$, and the radius is set as $R_{1}=0.2$ m. Therefore, the safe distance is calculated as $S_{1}=R_{1}+R_{L}=0.23$ m. For obstacle 2, the center coordinate is set as $O_{2}=\left[1.4,-0.5,-0.3\right]$, and the radius is set as $R_{2}=0.15$ m. Therefore, the safe distance is calculated as $S_{2}=R_{2}+R_{L}=0.18$ m.

**Figure 23:**
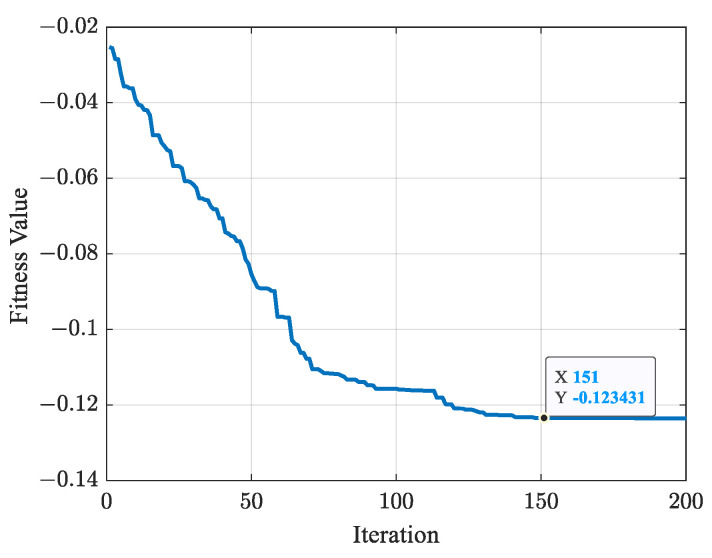
Variation curve of fitness values.

**Figure 24:**
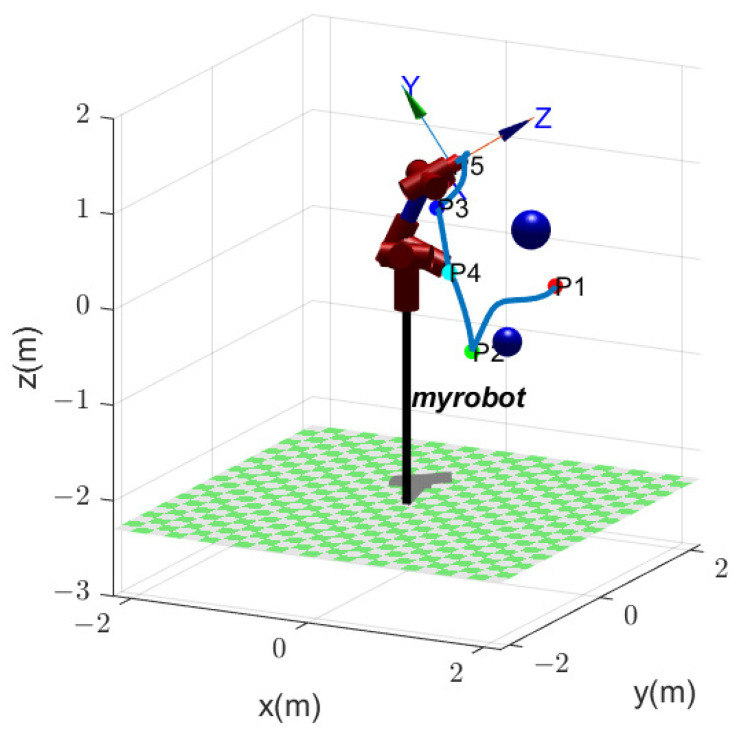
EE path with two obstacles.

Figure 23 shows the variation in fitness values of cost function $f_{K}$ in (12). After 151 iterations, the optimal fitness value is obtained as $f_{K}=-0.123431$, with the optimal parameter K=[3.3697,−9.2808,0.5973,−7.7359,4.0443,−19.3236,1.0619]. The total joint angular variation is $f_{Q}=24.9417$ rad, and the total EE path length is $f_{L}=5.6012$ m. The EE path is shown in Figure 24, where the EE passes through the task points in the order of $P_{1}\to P_{2}\to P_{4}\to P_{3}\to P_{5}$.

**Figure 25:**
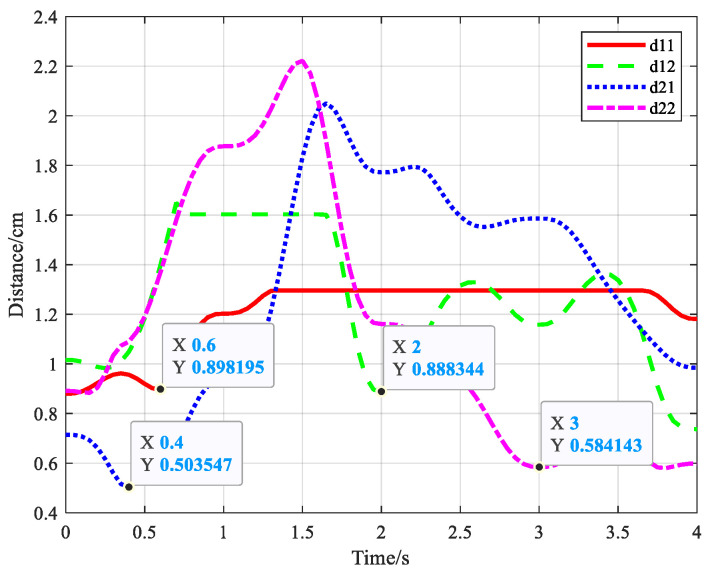
Distance variation between simplified S-R-S links and obstacle.

Figure 25 shows the variation in distance between obstacle i center and simplified link j. $d_{ij}$ denotes the distance, where i = 1, 2; j = 1, 2. For obstacle 1, the safe distance is $S_{1}=0.23$ m. The minimum distance between simplified obstacle center and link 1 is $d_{11}=0.898195$ m > $S_{1}$. The minimum distance between the simplified obstacle center and link 2 is $d_{12}=0.888344$ m > $S_{1}$. For obstacle 2, the safe distance is $S_{2}=0.18$ m. The minimum distance between the simplified obstacle center and link 1 is $d_{21}=0.503547$ m > $S_{2}$. The minimum distance between the simplified obstacle center and link 2 is $d_{22}=0.584143$ m > $S_{2}$. Therefore, the minimum distance between the simplified S-R-S manipulator and obstacle is larger than the safety distance. In conclusion, the manipulator has no collision with the obstacles during movements.

**Figure 26:**
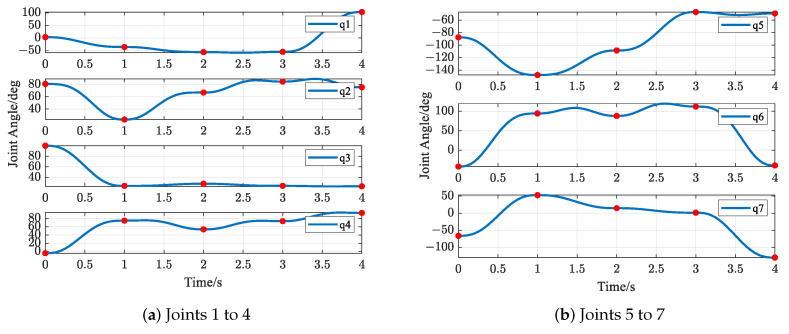
Profiles of angles in joint space.

**Figure 27:**
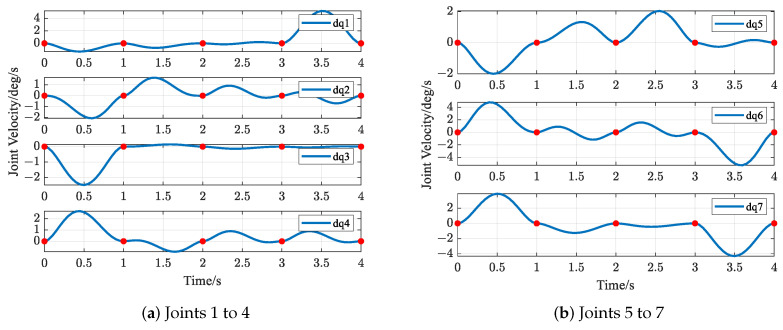
Profiles of angular velocity in joint space.

**Figure 28:**
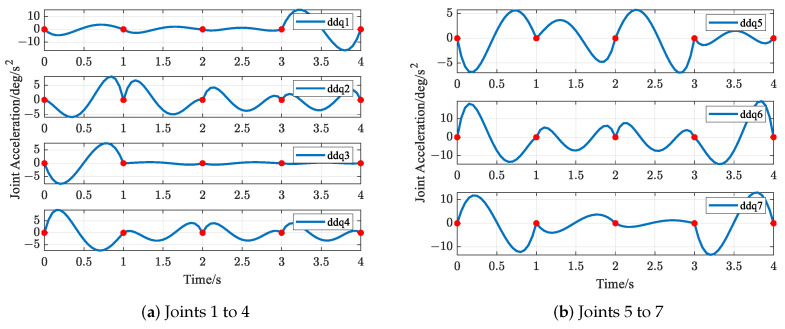
Profiles of angular acceleration in joint space.

**Figure 29:**
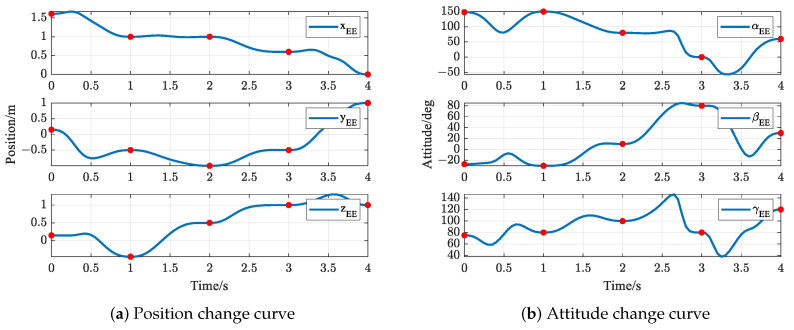
End-effector trajectory.

The trajectories of the S-R-S components are shown in Figure 26, Figure 27, Figure 28 and Figure 29, including the joints and the EE. The red solid points in the figures indicate the corresponding values at the task points. Figure 29 illustrates the trajectory of the end-effector. In Figure 29b, $\alpha_{EE}$, $\beta_{EE}$ and $\gamma_{EE}$ denote the Euler angles around the $X_{EE}$ axis, $Y_{EE}$ axis, and $Z_{EE}$ axis, respectively. In Figure 29, the end-effector reaches each task point exactly once and only once, and passes through the task points in the order of $P_{1}\to P_{2}\to P_{4}\to P_{3}\to P_{5}$. The variations in joint angles, anular velocities and angular accelerations are respectively shown in Figure 26, Figure 27 and Figure 28. As shown in Figure 26, the manipulator does not have violent movement, each joint angular curve is smooth and each joint angle is within the constraint range [−180,180] (deg). In Figure 27 and Figure 28, each joint angular velocity and each joint angular acceleration are zero at each task point. Therefore, the multitask-based anti-collision optimal trajectory of the S-R-S manipulator is derived, where two obstacles are considered.

The results of the above simulation experiments show that the SEGA algorithm performing multi-task obstacle avoidance is still effective for the case with multiple obstacles.

In theory, the proposed SEGA can also be employed for the case with multiple obstacles. Within the workspace of the manipulator, the obstacles can be successively detected according to Equation (2)–(4) in the part of Section 3. According to the detection results and the cost function in Equation (12), the manipulator trajectories are optimized utilizing the SEGA.

## 7. Conclusions

A direct solution method, the special encoding genetic algorithm (SEGA), is developed for multitask-based anti-collision trajectory planning of redundant manipulators. The trajectory planning problem is transformed into a mathematical optimization problem which is solved utilizing an SEGA. The method enables the manipulator to perform multiple tasks in one travel. The obstacles are successfully avoided, while the efficiency of the manipulator movement is improved. In the SEGA, each chromosome consists of three categories of genes, including the sequential order of task areas, the sequential order of joint configurations corresponding to task areas, and the unknown coefficients for anti-collision joint trajectories. The priority of the SEGA is validated through comparisons under MATLAB environments. The multitask-based anti-free-motion trajectories are generated in the joint space. The profiles of angular velocity and acceleration in joint space are continuous and smooth. Moreover, the trajectory length and angular variation are optimized. The effectiveness of the SEGA is further verified by considering the cases with multiple obstacles and different multitasking points. For further research, the proposed SEGA will be verified on an actual robot. Additionally, the multitask-based trajectory planning will be studied considering multiple dynamic obstacles.

## Figures and Tables

**Table 1:** D-H parameters of S-R-S manipulator.

Link i	$\theta_{i}\;{(}^{\circ })$	$\alpha_{i}\;{(}^{\circ })$	$a_{i}\;{(}^{\circ })$	$d_{i}$ (m)
1	−90	90	0	0.3
2	180	90	0	0
3	0	−90	0	0.7
4	0	90	0	−0.3
5	0	−90	0	0.7
6	0	90	0	0
7	−90	0	0	0.3

**Table 2:** Position and attitude of the five task points.

Task Point	Position/m	Attitude/deg
$P_{1}$	[1.61; 0.15; 0.15]	[148; −27; 75]
$P_{2}$	[1.00; −0.50; −0.45]	[150; −30; 80]
$P_{3}$	[1.50; 0.00; 0.00]	[100; 0; 100]
$P_{4}$	[1.00; −1.00; 0.50]	[80; 10; 100]
$P_{5}$	[0.00; 1.00; 1.00]	[60; 30; 120]

## Data Availability

Data are contained within the article.
